# Developmental Programming of the Fetal Immune System by Maternal Western-Style Diet: Mechanisms and Implications for Disease Pathways in the Offspring

**DOI:** 10.3390/ijms25115951

**Published:** 2024-05-29

**Authors:** Benjamin N. Nelson, Jacob E. Friedman

**Affiliations:** 1 Harold Hamm Diabetes Center, University of Oklahoma Health Sciences Center, Oklahoma City, OK 73104, USA; benjamin-n-nelson@ouhsc.edu; 2 Department of Physiology and Biochemistry, University of Oklahoma Health Sciences Center, Oklahoma City, OK 73104, USA; 3 Department of Pediatrics, Section of Diabetes and Endocrinology, University of Oklahoma Health Sciences Center, Oklahoma City, OK 73104, USA

**Keywords:** maternal obesity, fetal–maternal health, innate immune regulation, microbiome, metabolites, epigenetics, multi-omics

## Abstract

Maternal obesity and over/undernutrition can have a long-lasting impact on offspring health during critical periods in the first 1000 days of life. Children born to mothers with obesity have reduced immune responses to stimuli which increase susceptibility to infections. Recently, maternal western-style diets (WSDs), high in fat and simple sugars, have been associated with skewing neonatal immune cell development, and recent evidence suggests that dysregulation of innate immunity in early life has long-term consequences on metabolic diseases and behavioral disorders in later life. Several factors contribute to abnormal innate immune tolerance or trained immunity, including changes in gut microbiota, metabolites, and epigenetic modifications. Critical knowledge gaps remain regarding the mechanisms whereby these factors impact fetal and postnatal immune cell development, especially in precursor stem cells in bone marrow and fetal liver. Components of the maternal microbiota that are transferred from mothers consuming a WSD to their offspring are understudied and identifying cause and effect on neonatal innate and adaptive immune development needs to be refined. Tools including single-cell RNA-sequencing, epigenetic analysis, and spatial location of specific immune cells in liver and bone marrow are critical for understanding immune system programming. Considering the vital role immune function plays in offspring health, it will be important to understand how maternal diets can control developmental programming of innate and adaptive immunity.

## 1. Introduction

Obesity is a world-wide epidemic that impacts human health at all ages, including pregnant women and their offspring, with socioeconomic consequences across generations in both developed and developing countries. While difficult to approximate, pan-global studies have estimated that about 500 million people are currently obese, and that number is expected to reach over 1 billion by 2030 [1,2]. With the rising global incidence of obesity, especially in children and women of reproductive age, it is important to understand how obesity and poor maternal diet modify neonatal outcomes, particularly in the first 1000 days from conception to 2 years of age. Critical developmental windows in a child’s life can have lifelong impacts on the structure and function of organs and tissues, including developmental programming of the immune system, which plays a significant role in disease susceptibility [3,4,5]. Maternal diet, particularly a western-style diet (WSD) high in fat and simple sugars, has been shown in animal models to drive developmental programming of the immune system, beginning in utero [6,7]. The underlying causes and mechanisms for this remodeling, its impact on future disease pathways, and the critical events that drive developmental programming in the immune system are just beginning to be investigated. The purpose of this review is to explore how exposure to excess maternal fuels during fetal life can influence innate immune cell development and responses, microbial dysbiosis, metabolic effects from metabolites, and epigenetic modifications. How these changes affect the development of mechanisms underlying long-lived chronic inflammatory diseases, such as metabolic dysfunction-associated steatotic liver disease (MASLD, formerly NAFLD), including microglial and liver function in the offspring, are reviewed. Possible interventions in pre-clinical models of disease are also discussed.

## 2. Immune Cell Development: Hematopoiesis

**Figure 1:**
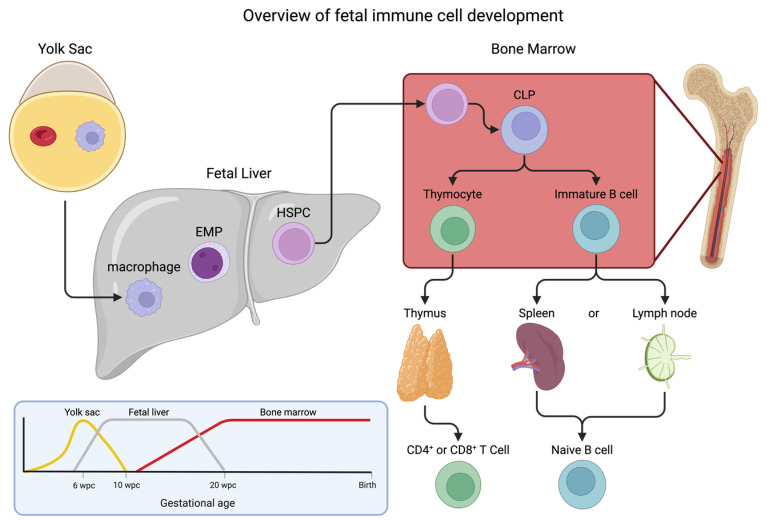
Simplified overview of fetal immune cell development. Immune cells develop in three overlapping waves: firstly, primitive macrophages emerge from the yolk sac and, secondly, erythro-myeloid progenitors (EMP) form in the fetal liver. During the second and third trimesters, fully functioning hematopoietic stem and progenitor cells (HSPC) seed the fetal liver and migrate from the liver to the fetal bone marrow. The relative contribution of each wave is plotted against fetal gestational age in the number of weeks post-conception (wpc) until birth. T and B cells require further development through primary and secondary lymphoid organs starting from the common lymphoid progenitor (CLP) in the bone marrow (simplified here). Circulating leukocytes are replenished from this central reservoir in the bone marrow.

From embryo to neonate, the developing fetus undergoes many changes to its immune system. To understand the context of these developmental changes to the innate and adaptive immune systems, we provide a description of the tissues and cell types involved in normal development. Central to this process, hematopoiesis undergoes significant changes in the bone and liver during fetal development to yield all blood cells which are derived from a group of self-renewing, multilineage hematopoietic stem and progenitor cells (HSPCs) [8]. However, the notion that there is a single progenitor cell has been replaced by a layered concept, in which the various immune cells arise from predecessor cells in three broadly defined waves (Figure 1) [9,10]. The first cells observed in an embryo are not HSPCs but rather erythrocytes that supply oxygen to the fetal yolk sac [11]. These erythrocytes differ from their adult cousins in that they are nucleated and express embryonic hemoglobin [12]. In addition to these embryonic red blood cells, primitive macrophages develop within the yolk sac that are distinct from circulating macrophages. These cells travel to the fetal liver (establishing a resident population of macrophages and mast cells) before migrating to the brain as microglial cells or the lung as alveolar macrophages [13,14]. This is usually considered the first wave (termed “primitive”), which begins at about 4-weeks post-conception, as these primitive macrophages are the first immune cells to emerge from the yolk sac [15]. The second wave, beginning around 6-weeks post-conception, comes in the form of erythro-myeloid progenitors which are generated from hemogenic endothelial cells [16]. These progenitors have the potential to differentiate into macrophages, monocytes, granulocytes, and mast cells [17,18,19]. Finally, around 10 to 12-weeks post-conception, the third wave, known as definitive hematopoiesis, occurs when adult-type HSPCs emerge from the aorta gonad mesonephros region and seed the fetal liver, followed shortly thereafter by colonization of the fetal bone marrow [20]. It has been shown that this seeding of the bone marrow by HSPCs happens as early as embryonic day 15.5 in mice models [21]. These cells have the potential to generate the full spectrum of immune cells needed for proper functions throughout life [22].

In addition to myeloid cells, common lymphoid progenitor cells go through a series of developments outside of the bone marrow during the maturation of a fetus [23]. Lymphoid cells are a diverse group of immune cells, from the adaptive responses of T and B cells to their innate partners, including natural killer (NK) cells and innate lymphoid cells. Lymphoid progenitors derived from progenitor cells in the yolk sac expand in the fetal bone marrow where they account for ~40% of the total progenitor pool [24,25]. Primary development of these tissue resident immune cells happens early in fetal gestation and, once seeded into tissues, changes to “central” hematopoiesis and has little effect on the peripheral systems. Interestingly, the organs required for normal development (bone marrow and thymus) are not fully formed at this time. Despite this, cells with a similar genetic profile as lymphoid-myeloid progenitors are found within the fetal liver, suggesting that the pathways for the development of T/B cells are present at this stage [26]. Development of T-cell receptor subtypes undergo increasing diversity as development proceeds starting with invariant γδ T cells and proceeding through the more complex αβ T cells [27]. While B cells are traditionally associated with adaptive immunity, where they produce antibodies in response to specific pathogens, they also play an important role in the innate immune system. Human B cell development is a dynamic lifelong process that starts in utero in the bone marrow at around 6-weeks post-conception [28]. B cell-mediated innate immunity is the body’s immediate, nonspecific defense mechanism against pathogens. B cells can interact with gut microbiota, which play a crucial role in metabolic health [29]. Naïve B cells also show lineage divergence depending on when the cells are seeded. Tissue resident B-1 cells can only be differentiated from fetal bone marrow or fetal liver; this contrasts with conventional B-2 cells, which are derived only from adult bone marrow [30].

## 3. Impact of Maternal Obesity/WSD on Maternal and Placental Immune Function

Normal pregnancy proceeds with many changes in maternal immune function and a highly regulated inflammatory response is required for proper development of the fetus [31]. Maternal obesity can have many negative influences on the regulation of immunity and inflammation over the course of gestation [32]. A majority of studies demonstrate that pregnant women with obesity exhibit increased inflammation and circulating inflammatory cytokines compared with normal-weight pregnant women, and factors including fuel overload, insulin resistance, and excess gestational weight gain contribute to this inflammation [33,34,35,36]. Pre-gravid obesity impacts the systemic maternal immune system, e.g., reducing the numbers of NK T cells and plasmacytoid dendritic cells (DCs) [37,38]. Additionally, changes to the adaptive immune cells have been observed. While the total number of CD4^+^ T cells remained constant, the number of naïve CD4^+^ T cells was reported to increase in plasma across gestation in obese pregnancies [37,38]. As for CD8^+^ T cells, studies on maternal obesity suggest an increase in their numbers from mid to late pregnancy, possibly as a response to the growing demands of the maternal–fetal interface and the need to maintain immune surveillance [37,38].

The placenta plays a crucial role by providing oxygen, nutrients, and immune protection to the developing fetus, as well as serving as an endocrine organ that regulates maternal–fetal communication and maternal physiology. The predominant immune cells in the placenta are the uterine NK (uNK) cells and macrophages (Hofbauer cells) [39,40,41]. Unlike normal NK cells, which are cytotoxic, uNK cells are generally responsible for angiogenesis in the placenta and maternal immune tolerance towards the fetus [42]. Additionally, Hofbauer cells play a role in morphogenesis and homeostasis and display an M2 phenotype (related to tissue repair and renewal for the growing fetus) by affecting the trophoblast cells [43]. Proper regulation of these cells is crucial for normal development of the fetal immune system and maternal obesity/WSD can alter this regulation. Extracellular vesicles can provide some of this regulation by way of membrane-bound proteins and miRNAs. However, the discussion surrounding fetal regulation by extracellular vesicles is an emerging field and has been reviewed elsewhere (see [44,45]).

### 3.1. Maternal Obesity Changes Placental Immunity

Whether maternal obesity leads to heightened cytokine transport across the placenta, thus directly inducing inflammation in the fetus, is an open question. However, one study revealed that fetal-derived IL-8 helps recruit maternal neutrophils in normal pregnancies [46]. Additionally, it has been shown that pregnancy increases systemic inflammation and pre-gravid obesity increases this further. Excess maternal adiposity has been reported to alter the composition and function of maternal monocytes in peripheral blood, as well as macrophages found in placental and maternal adipose tissues during late gestation, toward a proinflammatory phenotype [33,47]. A recent study showed that Hofbauer cells and microglia share a similar developmental lineage and correlations in gene dysregulation were observed between the populations during maternal obesity [48]. This suggests that the readily abundant cells in the placenta provide insight into the developmental function of neonatal microglia. Higher maternal BMI has also been correlated with increased numbers of B cells during pregnancy [38]. Furthermore, pregnant women with obesity exhibit increased activation of uNK cells promoting TNF-α production [49,50]. The normal cellular population of the placenta is also altered under obese conditions; an increased accumulation of CD68^+^ macrophages and a reduced number of M1 (proinflammatory) polarized macrophages have been reported [51,52]. Instead of inflammation, the accumulating cells in the placenta are hyporesponsive to TLR stimulation, which is associated with systemic and adipose tissue inflammation in obese pregnancies [53,54]. These cells at the maternal–fetal interface thus become excessively tolerant to stimuli and the tolerance is then imparted onto the fetus by shared developmental lineages.

### 3.2. Cord Blood Changes in Offspring from Obese Pregnancies

**Figure 2:**
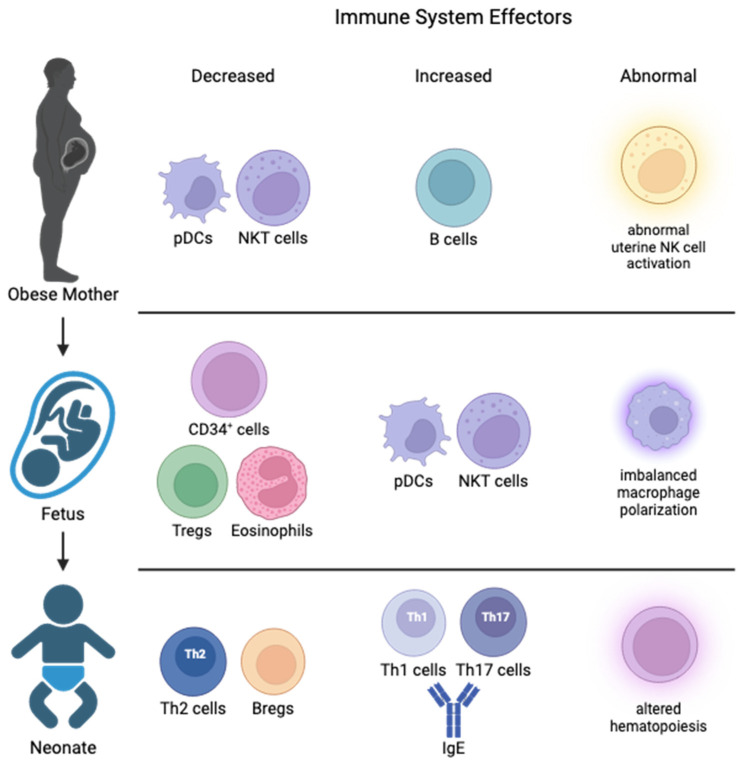
Changes to immune system effectors in the mother, fetus, and neonate due to maternal obesity. Maternal obesity is accompanied by increased inflammation and abnormal uterine natural killer (NK) cell activation. These changes alter the blood supply to the fetus by reducing fetal pluripotent CD34^+^ cells and regulatory capabilities and altering macrophage polarization. The neonate born to a mother with obesity may have imbalanced T helper subtypes, increased circulating IgE, and altered hematopoiesis leading to an increased chance of childhood allergies and autoimmune diseases. Bregs, B regulatory cells; NK, natural killer; NKT, NK T cells; pDCs, plasmacytoid dendritic cells; Th, T helper; Tregs, T regulatory cells.

Changes during pregnancy can have a profound impact on the immune cells and maternal-derived inflammatory cytokines, which can alter the developmental trajectory of the neonate (Figure 2). In normal human pregnancy, the fetal immune system has a helper T (Th) 2 phenotype to prevent alloimmune responses against the mother [55]. This is facilitated by a rare B cell subpopulation (regulatory B cells) and primarily by cytokines IL-10, IL-35, and TGF-β [56]. The immune tolerance function of these cells acts by inhibiting Th1 cell activation, Th17 differentiation, and maintenance of Tregs [57]. In cord blood from obese pregnancies, an increase of NK T cells and plasmacytoid DCs with a corresponding drop in CD34^+^ stems cells, eosinophils, and regulatory T cells (Tregs) have been shown [58]. This drop in hematopoietic stem cells may alter the host’s ability to combat a wide range of insults to the immune system and instead skew it towards predetermined remodeling. Indeed, Sureshchandra et al. found that memory T cells within cord blood from obese pregnancies were increased in number; however, the CD4^+^ T cells had functional defects and blunted responses to stimuli [59]. This diminished ability of T helper cells impacts their ability to produce IL-4 as it is negatively correlated with increasing maternal BMI [53]. Paradoxically, unusually high levels of inflammatory mediators, such as TNF-α and C-reactive protein, have been observed in cord blood from obese pregnancies [60,61]. All these changes lead to blunted responses in fetal immune cells, decreased placental blood flow/angiogenesis, and increased fatty acid uptake with a corresponding increase in fetal liver mass in obese gravida [62]. Further, ex vivo studies have shown that monocytes in cord blood from obese pregnancies exhibit increased susceptibility and severity to infections, which arose from aberrant monocyte responses caused in part by reduced nuclear translocation of NF-κB [7,51]. These studies showed that reduced availability of proinflammatory gene promoters led to decreased inflammatory mediators, such as IL-6, IL-10, and IL-12. Taken together, maternal obesity has the capacity to change both relative immune cell populations in the pregnant woman and in her fetus to an immune-tolerant phenotype that can impact inflammation pathways postnatally.

## 4. Impact of Maternal Obesity on the Fetal Liver and Offspring Immunity

MASLD is a chronic, progressive condition affecting about 37% of the global population and is strongly associated with features of the metabolic syndrome, including obesity and type 2 diabetes [63]. In animal models, maternal diet-induced obesity has been shown to modify fetal liver and immune system development and function [64,65]. Studies in rodents and nonhuman primates (NHP), in addition to human neonates, link maternal obesity with increased neonatal liver fat [66,67,68]. Further, exposure to a maternal WSD can increase the risk of the pediatric form of MASLD in adolescent youth [69]. Maternal factors that impact fetal liver development are a complex interplay involving pre-gravid obesity, dietary patterns, and inflammation. Maternal genotypes may influence the development of MASLD in offspring and thrifty genes that influence obesity may be involved. Candidate genes directly involved in obesity that affect energy balance (i.e., hypothalamic satiety receptor MC4R and adipo-genic transcription factor SREBP1 [70,71]) may contribute to genetic risks in the offspring.

MASLD can progress to its active inflammatory form, metabolic dysfunction-associated steatohepatitis (MASH), and both the innate and adaptive immune systems contribute to the progression [72]. Liver resident Kupffer cells respond to nutrient overload and liver damage by releasing chemokines CCL1, CCL2, and CCL5 to recruit monocytes and promote their polarization into M1 proinflammatory macrophages in a CCR8-dependent manner [73,74]. Chemically blocking these mediators has been shown to reduce hepatic fibrosis in human trials [75]. However, inflammation from the innate immune system is not the only driving force for increased complications in the liver, as there is an influx of T and B cells into the liver forming ectopic lymphoid structures [76]. During hepatic steatosis in mice, CD4^+^ T cells will produce IFN-γ from Th1 cells through the expression of T-bet, further increasing inflammation [77]. Studies using IFN-γ-deficient mice have shown decreased hepatic steatosis and fibrosis when compared with wild-type mice [78]. Similar findings have been corroborated in humans through clinical observations in both adults and children [79,80], suggesting that animal studies on the developmental effects of MASLD are translational to humans.

Maternal WSD can induce a proinflammatory response in fetal HSPCs and macrophages from NHPs [81]. Several adaptive programs have also been described in macrophages, including priming, tolerance, and trained immunity [82,83]. Specific pathways and mechanisms differ between the various adaptive programs in innate immunity, involving epigenetic, transcriptional, and metabolic reprogramming. Innate immune cells can undergo any of these functional adaptive programs, but the exact definition depends on the context under which the cells are studied and the nature of the stimulus used before and after to demonstrate an activated state of programmed immunity or inactivated state of immune tolerance to secondary stimuli [84,85]. Depending on their inflammatory polarization, macrophages in the liver either promote resolution from inflammation and prevent progression to fibrosis or remodel the tissue and provoke fibrosis. Their state of immune tolerance may affect their ability to expand in number, leading to cellular exhaustion in resident tissues in response to secondary challenges, such as infection or a WSD. Given that immune tolerance can lead to downregulation of inflammatory and restorative pathways in the liver, pathogenic immune tolerance may have broad consequences on increasing programming effects through susceptibility, initiation, or progression of fibrosis across a variety of tissues.

Fetal and neonatal T and B cells are not spared from changes to normal effector functions due to external stimuli. CD4^+^ and CD8^+^ T cells respond to antigens in HFDs to drive MASH and metabolic syndrome [77,86]. These cells are recruited into the liver by IFN-α and promote insulin resistance and glucose metabolism during HFD feeding [87]. Disruption of the gut microbiota-B cell axis may also contribute to obesity-related inflammation and metabolic dysfunction. B cells have also been implicated in the pathogenesis of MASLD, particularly in its more severe form MASH, as they are shown to be pro-fibrogenic in the form of plasma cells secreting IgA [88]. These are formed by proinflammatory mediators, which stimulate hepatic stellate cells into producing retinoic acid which is detected by the B cells [89]. Additionally, Odaka et al. found that mice born from dams on a high-fat diet (HFD) had increased IgE production [90]. Since IgE is one of the main effectors of allergy, maternal obesity may explain the increasing prevalence of childhood allergies. The adaptive arm of immunity can also be linked back to innate immunity. Independent of T cells, B-1 cells can secrete IgM natural antibodies, which can react with endogenous antigens, such as oxidized phospholipids [91]. These changes both increase the susceptibility to MASLD and drive its progression forward.

## 5. Microglia Activation by a Maternal WSD

Microglia are the resident immune cells of the brain and have similar functions to macrophages, such as immune defense and brain maintenance [92]. This group of self-renewing cells originate from the fetal yolk sac and any adverse effects on this population at birth can cause long-term consequences on brain function [93]. Many associations have been made between inflammation and neuropsychiatric disorders, including ADHD, autism, schizophrenia, and eating disorders [94]. Mattei et al. found that maternal immune activation with Poly(I:C) led to a downregulation of genes involved in the inflammatory response, microglial development, and phagocytosis in adult offspring [95]. However, genes involved in migration of microglial cells were upregulated, and these transcriptional changes could be reversed with treatment of minocycline, an antibiotic used as a possible treatment for schizophrenia [96]. Studies in an NHP model showed that offspring from dams with WSD-induced obesity have an inflammatory phenotype in the brain that includes increased microglial cell counts [97]. This study showed that, while pre-pregnancy adiposity was positively correlated with increased microglial cell counts, these counts were also negatively correlated with maternal WSD. The exact cause of these relationships is not entirely clear. However, further research by this group found that IL-12 was increased in the dams due to maternal WSD, which caused neurodevelopmental disorders in the offspring [98]. Another group confirmed the previous findings of decreased microglial cell counts due to maternal WSD but also showed that the deceased cell count did not persist one year postnatally [99]. This would suggest that neurodevelopmental trajectory and immune response is heavily dictated by early life events. Additionally, this activation is accompanied with the release of proinflammatory cytokines, e.g., TNF-α, IL-1β, and IL-6, which have been linked to neuronal loss, brain damage, and other neurodegenerative diseases [100]. For example, induction of the inflammasome through NLRP3 causes increased neuroinflammation and cognitive impairment and knocking out its receptor IL-1R1 can restore these deficiencies back to control levels [101]. This increased inflammation damages brain tissue and can alter the proper functioning of other resident cells. IL-6 was also found to be a critical regulator of microglial activation and it effects were abrogated either by knockout mice or neutralizing antibodies or increased by maternal IL-6 administration [102]. Lastly, IL-17A was also shown to increase microglial activation through direct administration to the fetal mouse brain [103].

## 6. Maternal Obesity Influences the Fetal Microbiome and Immune Development

Maternal diet alters the microbiome of both the mother and the offspring to regulate many processes, including gene expression, immune tolerance, and metabolism [104,105,106,107]. The maternal microbiome has been studied in great detail recently and studies have shown that differences in maternal diet have a profound impact on the diversity and taxa of the maternal and fetal microbiota [108,109,110,111,112,113,114,115]. Individual bacteria and their components, such as endotoxins, have been linked with changes in gut and circulating inflammatory cells, and these changes have been implicated in many chronic western diseases [104,116]. Data suggest that gross changes in abundances of obesity-enriched microbial genes are positively correlated with blood glucose levels in pregnant women with obesity and GDM and can be controlled by maternal diet, but the functionality of these changes remains unknown [117,118]. A portion of the maternal microbiome is vertically transmitted to the neonate, and early-life microbiota of the infant depends in part on the mode of delivery [119]. Infants born vaginally have a microbiome that resembles the mother’s vaginal and gut microbiota, while those born via cesarean section have a microbiome that resembles the mother’s skin microbiota and hospital environment [120]. These differences may have profound impacts on the long-term health of the infant, with research suggesting that offspring born via cesarean section are more prone to allergies, asthma, and obesity [121]. While the exact mechanisms are unclear, data show that newborns’ epigenetic landscape and immune responsiveness to microbes are due to the maternal prenatal immune status in mice, NHP, and possibly humans [6,122,123,124,125].

The process of neonatal colonization, whether it begins in utero, and the mechanisms involved are complex and not completely understood. Commensal microbes make their way to the infant through vertical transmission from the mother and their diversity is affected by perinatal conditions [126,127]. Research has shown that 72% of the neonate gut microbiome comes from the mother, but this number is substantially reduced to 25% in cases of recent maternal antibiotic use [128]. Infants exposed to antibiotics obtained antimicrobial resistant species mostly from the environment rather than through vertical transmission. This shows the potential dangers that can arise with nosocomial infections and antibiotic exposure in neonates. The hygiene hypothesis suggests that reduced diversity of early-life microbial exposure is associated with an elevated risk of allergic diseases later in life; however, studies have also linked susceptibility to allergic disorders to immune regulatory pathways triggered by some commensal microbes [129]. Soderborg et al. showed that, when microbes from 2-week-old infants born to mothers with obesity are transferred to germ-free mice, this imbalance disrupts myeloid cells (macrophage precursors) derived from the bone marrow and provokes obesity and a leaky gut leading to MASLD after a WSD challenge [130]. This metabolic rewiring of maturing macrophages is likely to be accompanied by epigenetic reprogramming that causes reduced bacterial phagocytosis and prevents liver inflammation. This may profoundly disrupt the establishment of the host–microbe symbiosis and suggests that there is a direct impact on offspring’s stem cells, which regulate immune development.

Concepts concerning the origins of developmental immune programming and how early life interactions can shape lifelong alterations and disease susceptibility through immune tolerance or trained immunity of the innate immune system have been reviewed by Hong and Medzhitov [131]. The first 1000 days of life are a critical period for proper development of a healthy gut, dominated by a diverse group of infant-type bifidobacteria, including *Bifidobacterium longum*, *B. bifidum*, and *B. breve* [132,133,134]. These species can metabolize human milk oligosaccharides that are abundant in breast milk and thus have a metabolic advantage in early colonization [135]. Breastfeeding has been correlated with reduced disorders, such as pathogen infection, autoimmune disorders, diabetes, and obesity [136,137]. Likewise, the acquisition of founder strains in the *Enterobacteriaceae* family in human newborns plays an essential role in development of normal neonatal immunity and susceptibility to metabolic diseases in later life. *Enterobacteriaceae*, as well as being a facilitative anaerobe, is a producer of lipopolysaccharide (LPS), important for trained innate and adaptive immunity, and is critical for normal immune tolerance [137]. Maternal diet can also shift the community of commensals [114,116]. An HFD can increase the number of anaerobes, such as *Bacteroidetes,* and obese individuals have a high *Bacteroidetes* to *Firmicutes* ratio; however, this is not a universal finding [138]. Table 1 lists key studies exploring the nature between the microbiome and disordered offspring immunity.

The microbiome can also have a wide range of effects on shaping and training the host immune system [144,145,146]. Normal colonization of microbiota has been shown to assist in the proper development of CD8^+^ T cell immunity through the priming of monocytes and macrophages, which release TNF that signals conventional DCs to produce the CD8^+^ T cell target IL-12p40 [147]. They can also promote early innate effectors of unconventional T cells, including mucosal-associated invariant T (MAIT) cells, NK cells, and γδ T cells [148]. Furthermore, commensals are necessary for promoting immune tolerance and creating proper immune defenses in the gut. Peripherally induced Tregs (pTregs) can support tolerance in the gut through RORγt while also abrogating Th17 responses [149,150]. Murine studies have shown that early life (2–3 weeks old) is a critical window for pTreg generation and failure to generate Tregs can lead to altered immune homeostasis and increased inflammatory intestinal diseases [151]. These alterations to immune signaling contribute to immunologic and metabolic disorders, which are normally prevented through proper priming of the immune system. This priming can occur as early as the second trimester; memory T cells were found in fetal lymph nodes, which can be activated by *Staphylococcus* and *Lactobacillus* [152]. Further, it has been shown that germ-free mice have compromised CD4^+^ T cell and Treg development, suggesting that microbial interaction is necessary for early immune system development [153]. Finally, serotonin was shown to be highly enriched during the first 3 weeks of life in the luminal contents from the gut of specific pathogen-free neonatal mice and is driven by the gut commensal microbiota to directly alter cell differentiation into Tregs [154].

Microbes can also directly influence the differentiation and effect of bone marrow HSPCs as the fetus develops. Studies have shown that mice lacking proper commensal microbes during development have low HSPC counts and defective myelopoiesis which resulted in impaired resistance to bacterial infections [155,156]. Recolonization of these mice with a healthy microbiota can rescue this phenotype via metabolites, microbial components, and nutritional support [157,158,159]. However, there is a complex interplay at work as not all stimuli provide the same outcome. An example of this was found in mice given different bacterial components and those supplemented with LPS had an increased pathogen killing capacity from myeloid cells compared with those given bacterial DNA instead [160]. The heterogeneity coincides with other studies that have found differences among monocytes in both mice and humans in that inflammatory environmental cues can influence the resulting population of monocytes [161,162]. These differences have lasting effects on both the myeloid cells and HSPCs [163]. Luo et al. found an overall decreased capacity for hematopoiesis with an altered microbiome brought on by HFD feeding in adult mice, and these changes were carried over by fecal transplantation of microbes from the HFD group to normal mice [164]. On the other hand, most studies of early life LPS result in dampening of innate immunity in monocytes in offspring from LPS-treated dams compared with offspring from control pregnancies, suggesting an impaired capacity to respond to infection. In infants born to mothers with chorioamnionitis, for example, monocytes have impaired production of inflammatory cytokines and lower expression of genes involved in antigen presentation and adaptive immunity [165,166]. Taken together, altered microbiomes can have a profound effect both on the proper development and maintenance of immunity during critical periods of fetal development.

## 7. Metabolites Regulate Fetal Metabolism and Immune Development

As noted earlier, infant-type *Bifidobacteria* species are well suited for colonizing the infant gut due to their ability to metabolize breast milk [132]. They produce a myriad of metabolites, including various types of short-chain fatty acids (SCFAs) and aromatic amino acids, which can directly regulate and alter host pathways (Table 2) [167,168,169,170]. The most common SCFAs in the gut are acetate, propionate, and butyrate, which are able to influence host processes through different mechanisms [171]. Butyrate is the preferred energy source for gut epithelial cells and can directly modulate genes by acting as histone deacetylases (HDACs) [172]. HDACs have been shown to suppress tumor growth in cancerous cells and have anti-inflammatory effects in healthy cells [173,174,175]. Moreover, butyrate supplementation in mammalian models has been shown to elevate beneficial gut microbiota, improve intestinal barrier function, and promote fat mobilization and utilization, resulting in the attenuation of hepatic steatosis through STAT3 signaling and HDACs [176,177,178]. Acetate and propionate are activators of G protein-coupled receptors and thus promote lipid and glucose metabolism in white adipose tissue [179,180]. Chen et al. found that maternal supplementation with propionate ameliorated intra-uterine growth restriction by downregulating glucose and lipid metabolism caused by hypoxia [181].

Another class of metabolites important for regulating inflammation are derived from the essential amino acid tryptophan (Table 2). Tryptophan regulation and utilization are important during pregnancy and are essential for proper fetal growth [182]. Tryptophan metabolites are derived from gut metabolism and are altered in obesity; higher amounts of the tryptophan product kynurenine and lower amounts of serotonin are associated with higher BMI and visceral fat mass [183,184]. Gut commensals metabolize dietary tryptophan to produce metabolites capable of modulating the host immune system, thereby linking the gut microbiota with nutrition, metabolism, and the innate immune response. The indole derivatives tryptamine and indole-3-acetate were shown to suppress the inflammatory responses of macrophages and reduce lipid production in hepatocytes [185]. Other tryptophan metabolites, e.g., indole-3-ethanol, indole-3-pyruvate, and indole-3-aldehyde, can prevent gut permeability by maintaining tight junctions [186]. Finally, indole-3-carbinol can decrease adipogenesis, thermogenesis, and inflammation by rescuing key transcription factors that become dysregulated by HFD feeding [187]. Central to most of these pathways is the aryl hydrocarbon receptor (AHR); many tryptophan metabolites are AHR ligands [188]. AHR signals the transcription factors NRF2 and NF-κB and has been shown to play a role in the progression of MASLD [189]. Ghiboub et al. reviewed the ongoing research into therapeutic treatments using tryptophan, including direct supplementation, microbiota-derived supplementation, and AHR ligand activation [190].

**Table 2:** Common metabolites from commensal microbiota, their mechanism of action, and the effect on the host.

Metabolite	Mechanism	Effect	References
Acetate	FFAR2 and acetyl-CoA carboxylase	Promoted lipid metabolism and reduced appetite	[191,192]
Propionate	FFAR2 and FFAR3	Promoted glucose metabolism	[191]
Butyrate	FFAR3	Anti-inflammatory effects and promoted lipid metabolism	[191]
Indole-lactic Acid	AHR and HCA_3_	Improved gut health and immune responses	[193]
Phenyllactic Acid	Mineral utilization	Increased lymphoid cell count without infection	[194]
4-Hydroxyphenyllactic Acid	DCAR-1	Triggered increased innate immune responses	[195]
Tryptamine	AHR	Suppressed inflammation	[185]
Indole-3-acetate	AHR	Suppressed inflammation	[185]
Indole-3-ethanol	MyoIIA, ezrin	Maintained gut permeability	[186]
Indole-3-pyruvate	MyoIIA, ezrin	Maintained gut permeability	[186]
Indole-3-aldehye	MyoIIA, ezrin	Maintained gut permeability	[186]
Indole-3-carbinol	UCPs, PPAR, sirtuin-1, leptin, aP2	Decreased adipogenesis, thermogenesis, and inflammation	[187]

## 8. Role of Epigenetic Programming in Immune Cells from Offspring Exposed to WSD

Epigenetic changes due to maternal diet/obesity can occur throughout neonatal tissues and cells with lasting impact on neonatal development and beyond. Recent studies have shown that cord blood DNA methylation in neonates born to mothers with obesity correlated with maternal triglycerides and childhood adiposity [196]. In NHP studies, both HSPCs and bone marrow-derived macrophages (BMDM) from maternal WSD-exposed 3-year-old offspring were epigenetically modified to a more proinflammatory signature [6], despite weaning at 7 months to a normal chow diet. More open chromatin (higher transcription) in regions promoting glycolysis and more closed chromatin in regions relating to oxidative phosphorylation were observed in fetal mononuclear cells, suggestive of immune cell chromatin remodeling by exposure to maternal WSD. These progeny cells were then able to transform into macrophages with an increase in genes that mark early development of proinflammatory macrophages, such as FOS/JUN, NF-κB, C/EBPβ, and STAT6 [6]. These findings indicate that maternal WSD led to a classic shift, increasing glycolytic metabolism that favors the proinflammatory M1 cell type [6]. This effect may also be further modified by the tissue microenvironment which affect their proper function [197,198]. Suter et al. showed that, in NHPs, maternal WSD can alter the expression of *NPAS2* (regulator of circadian genes in peripheral organs) in the offspring liver through histone modification at the promoter regions of genes favoring liver lipid deposition [199,200].

Maternal overnutrition also causes epigenetic changes within the hypothalamus of the fetal and postnatal brain [201,202,203]. Mouse studies showed that maternal WSD alters the arcuate nucleus region that controls NPY and AgRP and the appetite suppression neurons of POMC and CART [204]. Maternal overnutrition can increase the expression of NPY/AgRP while reducing POMC in the adult offspring’s brain [205,206]. Overall, this led to less sensitivity to the appetite suppression hormones leptin and insulin [205,207,208,209]. Specifically, hypermethylation occurs at the enhancer and promoter regions for POMC [210,211]. Interestingly, hypermethylation of the enhancer regions (but not the promoter) persisted into adulthood showing the prolonged programming effects of overnutrition [210]. Modulation of genes can also occur though HDACs. Suter et al. showed in the NHP model that a maternal WSD increased acetylation of histone H3 through the downregulation of the lysine deacetylase SIRT1 in the liver [212]. A study in mice showed that, in response to an HFD, *Hdac5* and *Hdac8* levels were increased in the hypothalamus, suggesting that they also play a role in gene expression under different metabolic conditions [213]. These alterations to the environment can alter the local hypothalamic region and, coupled with the proinflammatory response of activated microglia and astrocytes, suggests that metabolic reprogramming during gestation will drive microglia innate immune memory to shape their immune reactivity, leading to abnormalities of the brain in areas of both appetite stimulation and suppression [214,215].

## 9. Conclusions and Future Directions

**Figure 3:**
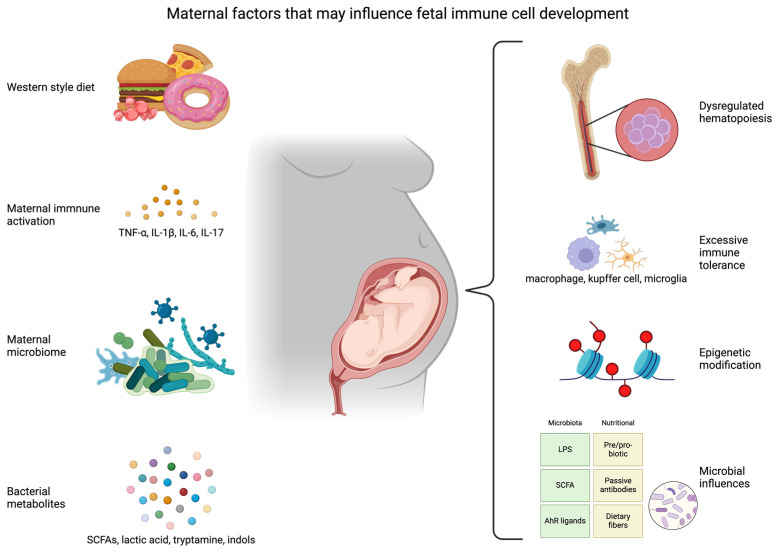
Maternal factors underlie developmental programming and immune cell development. Several maternal factors brought on by a maternal WSD include increased immune activation, altered microbiome, and changes to bacterial metabolite composition. These exposures underlie dysregulated hematopoiesis, excessive immune tolerance, epigenetic modifications, and disordered infant microbiome. The outcomes affect postnatal developmental programming in organs and tissues with sustained and long-term metabolic and neurodevelopmental effects on future health.

As outlined in this review, due to maternal factors, the developing immune system in offspring goes through many changes, both functionally and superficially in development, beginning with the fetus, to the neonatal period and beyond (Figure 3). However, many challenges remain as the interpretation and translation of these findings are hampered by our relatively limited mechanistic understanding of immune programming. As single-cell omic technologies are maturing and more high-throughput data analyses are conducted, more information is being gathered on different cell populations, especially immune cell types, rare cell types, disease state cells, and the whole spectrum of cells in between, which lead to complex interactions within tissues [216]. The signals underlying development of HSPCs, specifically in the fetal liver and bone marrow under the influence of maternal WSD, and the timing and distribution of cells are of critical importance. Tools with single-cell resolution for analyses of the transcriptome, epigenetic changes, splice variants, surface protein expression, and spatial location will enable us to uncover their interactions. Analyzing these types of datasets in tandem will allow us to discover novel interactions. Additionally, despite evidence that T and B cells can be derived from the yolk sac in mice, no definitive proof of this has been reported in humans, as fate-mapping in adults from their developmental origins is not yet possible [23]. Which HSPC populations function primarily during fetal development and which populations are lifelong will be key to understanding how early factors influence disease development.

Maternal proinflammatory dietary factors, including high fat/high sugar, can alter the microbiome of both the mother and their offspring and the metabolites these microbes produce. However, the critical roles of a complex microbiome in influencing HSPCs and fetal programming/immunity are needed. The exact composition of the human maternal microbiota that is transferred to their offspring is vastly different between individuals. Therefore, protocols and mouse models mimicking the human condition for discovery of these relationships during critical windows of development need to be refined. Alterations in the maternal microbiome can lead to functional changes in offspring immune cell populations; this research will generate a diverse array of datasets, including transcriptomics, proteomics, metabolomics and, integrated together, meta-omics, to create a functional understanding of the processes of fetal immune development. This information can better inform us of the treatments needed and pre/probiotic usage during pregnancy for healthier outcomes.

Lastly, questions remain as to how to correct the immune system, especially if we medically supplement with an external source. Studies have sought to use maternal diets as an intervention for altered immunological programming and neural defects in offspring [217]. Will it be possible in the future to introduce targeted genetically modified immune cells as therapy for metabolically rewiring the immune system [218,219]? Given the increasing numbers of children and adolescents suffering from metabolic and behavioral disorders, and the role that inflammation may play in triggering the origins of disease, understanding the relationship between poor maternal diets and developmental programming of the immune system during early life may play a crucial role in maintaining the delicate balance between health and disease in the 21st century.

## Figures and Tables

**Table 1:** Maternal obesity impacts commensal microbiota and immunity in the offspring.

Subjects (Year)	Physiological Outcomes	Programming Effects	Reference
222 human infants and NOD mice (2016)	*Bacteroides* spp. are highly abundant in the at-risk human group *E. coli* LPS decreases frequency of diabetes compared to *B. dorei* LPS in mice	Early microbiota trains innate immune signaling and tolerance Different LPS subtypes can alter autoimmunity through immune system modulation	[137]
Germ-free mice colonized with microbes of infants from obese or NW mothers (2018)	Higher amounts of SCFAs and increased gut permeability Macrophages were hyporesponsive to LPS and showed reduced phagocytic capability	Microbes were able to induce metabolic and inflammatory changes in the liver and bone marrow Mice were susceptible to increased weight gain and fatty liver after a WD challenge	[130]
17,055 neonates from the general population (2019)	Increased risk for T1D was correlated with haplotypes DR3 and/or DR4 *Intestinibacter* and *Romboutsia* are associated with protective haplotypes	Risk for T1D is correlated with core microbiome and diversity Probiotic treatment may be suitable due to conserved antigen among taxa present in high-risk individuals	[136]
Antibiotic-treated and germ-free mice (2019)	Myeloid progenitors reduced in antibiotic-treated mice Commensals primed JAK signaling to promote TLR-enhanced myelopoiesis	Host microbiota primes TLR-enhanced myelopoiesis Regulation of myelopoiesis required for sustained and proper immune responses	[139]
46 full-term neonates from obese mother with and without GDM (2020)	Microbes involved in immune suppression increased in GDM group SCFA levels and microbial relative abundances were correlated	Innate immunity was reprogrammed by altered microbiota Changed to gut microbiota can influence host metabolism	[140]
418 mothers and their neonates (2021)	Reduction in alpha diversity in neonates from women with GDM including increased *Firmicutes* and decreased *Proteobacteria* Metabolites indicate increased taurine/hypo-taurine, pyrimidine, beta-alanine, and bile acid metabolism	An increase in opportunistic pathogens may cause childhood metabolic disorders Pathways associated with increased metabolic function and fuels which may facilitate the development of childhood obesity	[141]
394 pregnant women in the first trimester and germ-free mice (2023)	Increased levels of IL-4, IL-6, IL-8, GM-CSF, and TNF-α and decreased amounts of SCFAs in women who later developed GDM Decreased *Prevotella* in women who later developed GDM	GDM can be predicted as early as the first trimester Markers for GDM diagnosis can include inflammation and microbiota Microbial composition drove inflammation and insulin resistance	[142]
65 mother-infant pairs, half receiving a prebiotic (2024)	Prebiotics were associated with decreased *Negativicutes* in mothers and infants Significant increases of the SCFA acetic acid were found in the prebiotic mother group	*Negativicutes* were correlated with c-section births and are less competitive in a prebiotic-rich nutritional environment Acetate is likely due to increased abundance of *Bifidobacterium*	[143]

GDM, gestational diabetes mellitus; LPS, lipopolysaccharide; SCFA, short-chain fatty acid; T1D, type 1 diabetes.
